# Arthroscopic anatomy of the posterolateral corner of the knee: anatomic relations and arthroscopic approaches

**DOI:** 10.1007/s00402-021-03864-6

**Published:** 2021-03-22

**Authors:** Jannik Frings, Sebastian Weiß, Jan Kolb, Peter Behrendt, Karl-Heinz Frosch, Matthias Krause

**Affiliations:** 1 Department of Trauma and Orthopaedic Surgery, University Medical Center Hamburg-Eppendorf, Martinistrasse 52, 20246 Hamburg, Germany; 2 Department of Orthopaedic and Trauma Surgery, University Medical Center Schleswig-Holstein, Kiel, Germany; 3 Department of Trauma Surgery, Orthopaedics and Sports Traumatology, BG Hospital Hamburg, Hamburg, Germany

**Keywords:** Posterolateral corner, Reconstruction, Anatomy, Arthroscopy, Preparation, Footprint

## Abstract

**Introduction:**

Although open-surgical techniques for the reconstruction of the posterolateral corner (PLC) are well established, the use of arthroscopic procedures has recently increased. When compared with open surgical preparation, arthroscopic orientation in the PLC is challenging and anatomic relations may not be familiar. Nevertheless, a profound knowledge of anatomic key structures and possible structures at risk as well as technical variations of arthroscopic approaches are mandatory to allow a precise and safe surgical intervention.

**Materials and methods:**

In a cadaveric video demonstration, an anterolateral (AL), anteromedial (AM), posteromedial (PM) and posterolateral (PL) portal, as well as a transseptal approach (TSA) were developed. Key structures of the PLC were defined and sequentially exposed during posterolateral arthroscopy. Finally, anatomic relations of all key structures were demonstrated.

**Results:**

All key structures of the PLC can be visualized during arthroscopy. Thereby, careful portal placement is crucial in order to allow an effective exposure. Two alternatives of the TSA were described, depending on the region of interest. The peroneal nerve can be visualized dorsal to the biceps femoris tendon (BT), lateral to the soleus muscle (SM) and about 3 cm distal to the fibular styloid (FS). The distal attachment of the fibular collateral ligament (FCL) can be exposed on the lateral side of the fibular head (FH). The fibular attachment of the popliteofibular ligament (PFL) is exposed at the tip of the FS.

**Conclusion:**

Arthroscopy of the posterolateral recessus allows full visualization of all key structures of the posterolateral corner, which provides the basis for anatomic and safe drill channel placement in PLC reconstruction. A sufficient exposure of relevant anatomic landmarks and precise portal preparation reduce the risk of iatrogenic vascular and peroneal nerve injury.

**Supplementary Information:**

The online version contains supplementary material available at 10.1007/s00402-021-03864-6.

## Introduction

Posterior instabilities of the knee are frequently associated with instabilities of the posterolateral corner (PLC) [1]. The PLC comprises the fibular collateral ligament (FCL), which stabilizes mainly against varus stress, and the posterior- and posterolateral stabilizing popliteus complex, which itself consists of the popliteus muscle/tendon unit (PLT), the popliteofibular ligament (PFL), the fabellofibular ligament and the popliteomeniscal fibers [arcuate complex (AC)] [2]. PLC injuries may lead to an increased posterior, external rotational and/or varus instability and, thus, provide the biomechanical basis for graft failure following cruciate ligament reconstruction [3–7]. Accordingly, anatomic reconstruction techniques have been proposed to stabilize the PLC better than non-anatomic techniques [8–10]. Recently, arthroscopic procedures have gained popularity for the treatment of posterolateral instabilities [11]. Some targeted techniques, such as the arthroscopic popliteus bypass, are designed to address isolated rotational instabilities (e.g. Fanelli type A) [12–14]. Other technical descriptions have focused on arthroscopic techniques for complex anatomic PLC reconstruction [15–18]. Although these techniques show promising biomechanical and clinical results, they are technically demanding and require a fundamental understanding of the arthroscopic posterolateral anatomy [14, 18]. Arthroscopic anatomy can be confusing and complicate the identification of relevant structures, as one can get lost easily in the “dark side of the knee”. In addition, there is a considerable fear of peroneal nerve or vascular injury, during preparation or tunnel placement [19].

This cadaveric video demonstration is intended to provide useful knowledge of anatomic proportions, landmarks and critical zones in the arthroscopic preparation of the PLC. A thoughtful choice of portals is crucial for successful preparation, drill channel placement and, thus, for successful treatment.

## Surgical technique

The following steps of arthroscopic preparation (Video 1) were performed by a senior physician (M.K.) with a large surgical experience of open PLC reconstruction and profound routine in arthroscopic surgery.

**Fig. 2:**
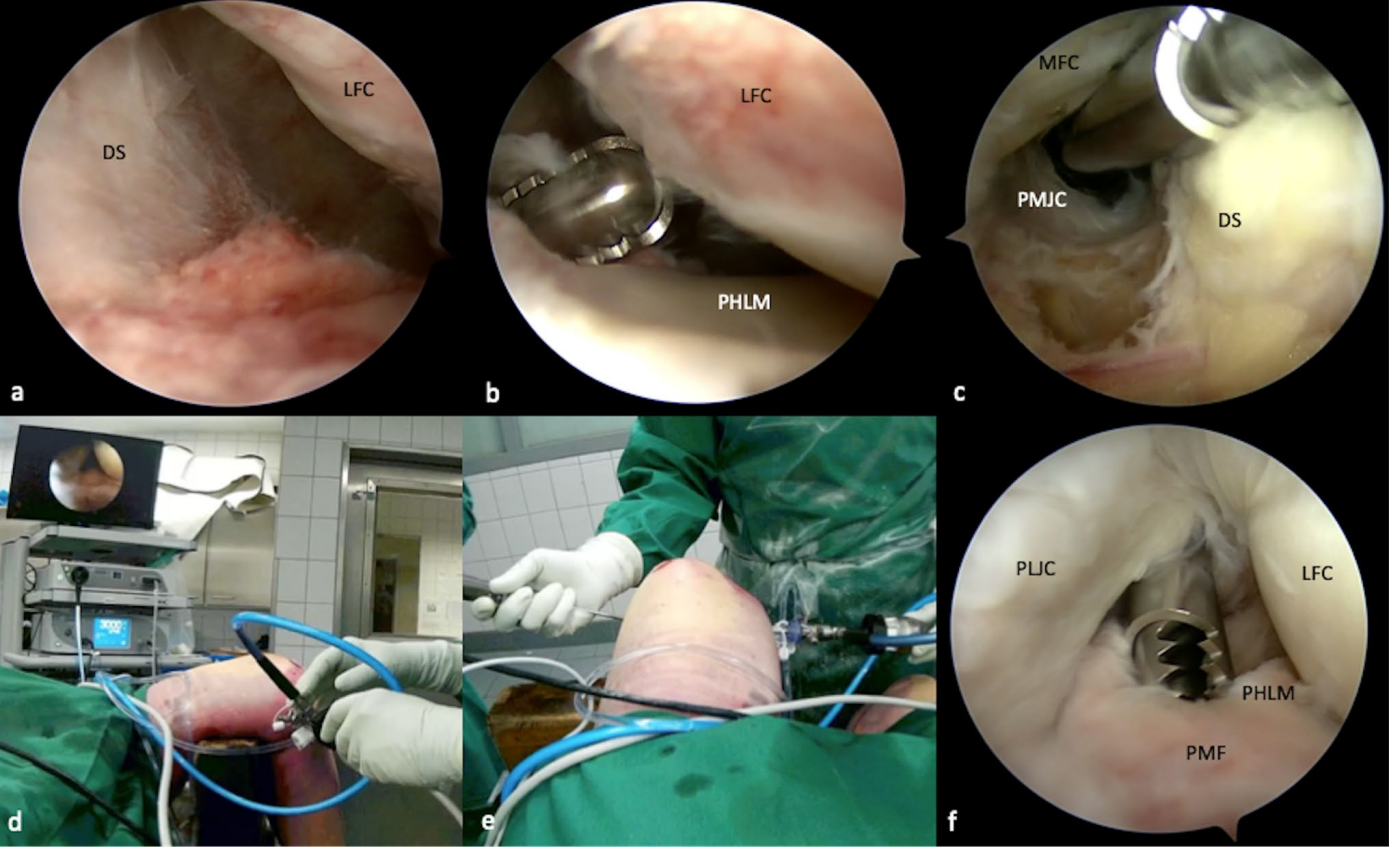
Transseptal approach and posterolateral portal. After implementing the posteromedial portal, the camera is retracted and passed underneath the anterior cruciate ligament and into the PL recessus visualizing the dorsal septum from the lateral side (**a**). A shaver (Excalibur®, Arthrex, Naples, FL, USA) is introduced through the posteromedial portal to create a transseptal approach under indirect visualization (**b**). Through the transcondylar view, size and position of the transseptal portal can be validated (**c**). To access the PL recessus, the arthroscope is placed into the PM portal and passed through the transseptal approach (**d**). Under direct visualization a PL portal is created, analogously to the PM portal (**e**, **f**). In case of revision surgery with extensive scarring or decreased opening of the medial joint space, primary visualization of the dorsal septum from the medial side may be required. Therefore, the arthroscope is first passed through the AL portal, underneath the PCL and into the posteromedial recessus, in order to observe resection of the septum. *DS* dorsal septum, *LFC* lateral femoral condyle, *PHLM* posterior horn of the lateral meniscus, *MFC* medial femoral condyle, *PMJC* posteromedial joint capsule, *PLJC* posterolateral joint capsule, *PMF* popliteomeniscal fibers
  In the case of revision surgery, posterior scaring, or limited lateral opening, a transcondylar intercruciate approach might be required. Once the transseptal portal is created, the arthroscope is switched to the PM portal, passed through the dorsal septum and into the PL recessus (Fig. 2d).

- Step 1: Diagnostic arthroscopy and transcondylar approach
  For arthroscopic visualization of the PLC, a minimum of four standard portals and one accessory approach are required: high anterolateral (AL), anteromedial (AM), posteromedial (PM), posterolateral (PL) portal and a transseptal approach (TSA).
  After the implementation of the AM and AL portal a diagnostic arthroscopy is performed and a PM portal is established (Video 1). Subsequently, the PM portal is used for establishment of the TSA and further preparation of the posterolateral recessus. To visualize the posterolateral recessus, the arthroscope can be passed either underneath the anterior cruciate ligament (ACL) or through the TSA from posteromedial.
- Step 2: The posteromedial portal
  Starting from a high AL portal the arthroscope is passed through the intercondylar notch and underneath the PCL into the posteromedial recessus (Fig. 1a, b). Under arthroscopic visualization a needle is inserted and a blunt perforation is performed after careful superficial skin incision in order to protect the saphenous nerve (Fig. 1c) [20]. Before incising the joint capsule, needle probing should be performed, in order to test the accessibility and direction, provided by the posteromedial portal. Placing the portal too far anterior might limit instrument angulation for the TSA and preparation of deep aspects of the posterolateral recessus as well as visualization of the distal structures of the PLC. In order to facilitate the following steps and maneuvers and to preserve the PM portal despite of continuous swelling, the use of a cannula (e.g. Twist-In®, Arthrex, Naples, FL, USA) can be recommended at this point (Fig. 1d).
- Step 3: Development of the transseptal approach (TSA)
  **Fig. 1:**
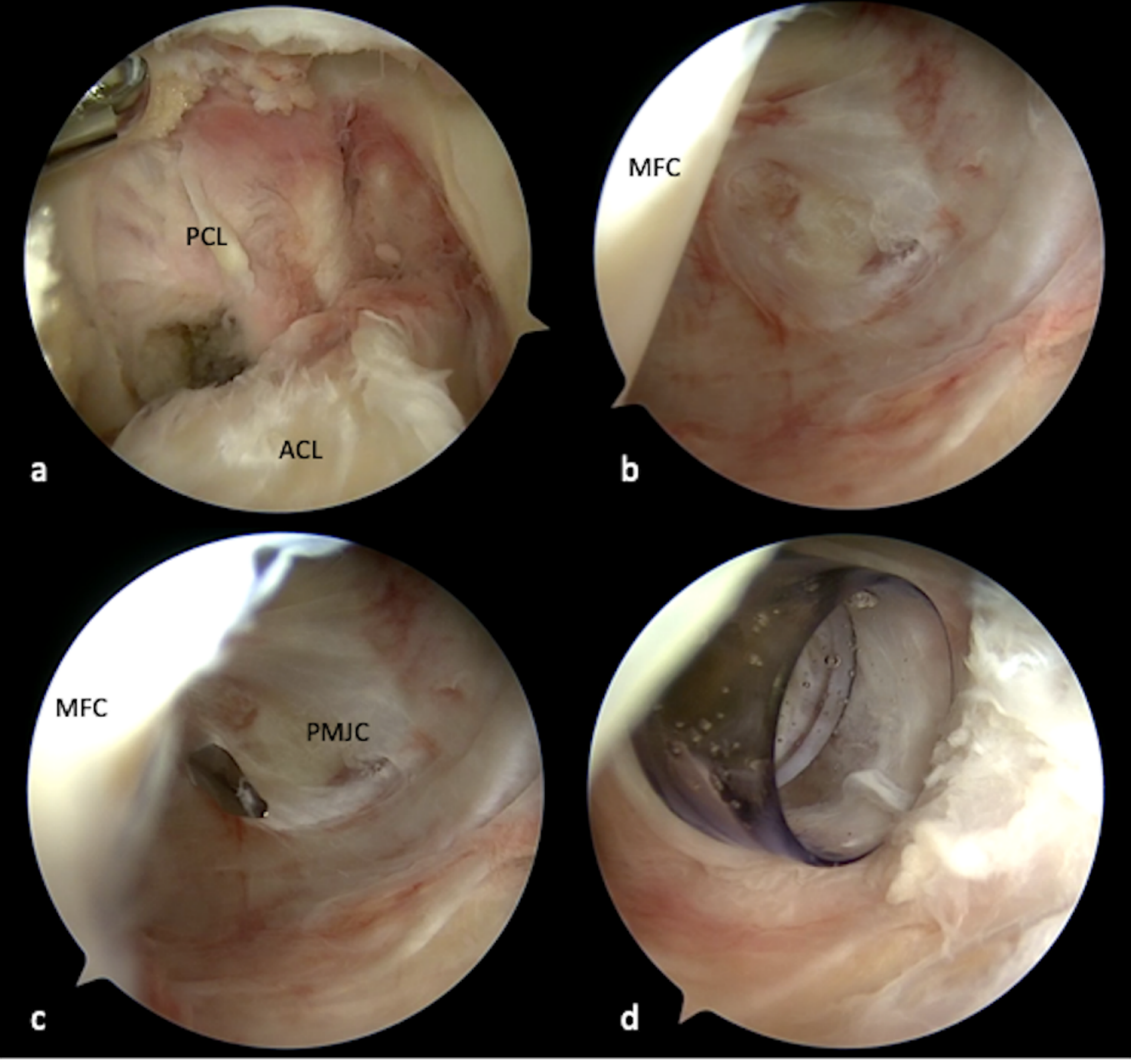
Transcondylar approach and implementation of the posteromedial portal in a left knee After preparation of the intercondylar notch, the arthroscope is passed underneath the posterior cruciate ligament into the posteromedial recessus (**a**), to visualize the posteromedial joint capsule (**b**). A needle is introduced for proper placement of the PM portal (**c**). Using a cannula (Twist-In®, Arthrex, Naples, FL, USA) can be helpful to secure the portal and facilitate management of instruments (**d**). *PCL* posterior cruciate ligament, *ACL* anterior cruciate ligament, *MFC* medial femoral condyle, *PMJC* posteromedial joint capsule
  The arthroscope is now placed into the AM portal and passed underneath the ACL to access the PL recessus. During this step, the lateral opening can be increased by applying varus stress to the flexed knee and thereby allowing easy intercondylar passage. The dorsal septum is now carefully resected with a shaver (e.g. Excalibur®, Arthrex, Naples, FL, USA) coming from the posteromedial portal while the septum is indirectly visualized from the lateral side (Fig. 2a–c). Alternatively, the arthroscope can be passed through the AL portal and underneath the PCL into the PM recessus to gain direct visualization of the medial side of the dorsal septum during resection. Thereby, however, preparation must not be extended too far distally to protect the popliteus muscle and fibers of the posterior cruciate ligament (PCL) during resection.

### Strategic considerations of the TSA

Specific indications may require different variations of the transseptal approach. In the presented case, a low transseptal approach was created and reconfirmed by direct verification of proper placement. Thereby, an exact placement of the portal is possible and (posterior or distal) misguidance of the shaver during preparation can be prevented (Fig. 2b, c).

### Anatomic relations of popliteal vascular structures

The popliteal artery (PA) is the most anterior structure of the popliteal neurovascular bundle [21]. At the joint line level, it is located adjacent to the dorsal septum, posterior and slightly lateral (2–3 mm) to the PCL [21–23]. Distally, the distance between PA and posterior capsule decreases and is smallest at about 1 cm below to the joint line, where it is retained by the fibrous arc of the soleus muscle [21, 22]. Accordingly, there is a considerable risk of injury during posterior knee arthroscopy and in particular during preparation of the TSA or drilling of tibial tunnels [24, 25].

Depending on knee positioning during arthroscopy, this risk can be reduced significantly. With progressive knee flexion, the posterior clear space increases, as the PA moves posteriorly [26]. During arthroscopy in full extension, its distance to the tibial PCL insertion is 5.4 mm, but almost twice as much in 90–100° of flexion (9.7–9.9 mm) [26, 27]. In the same position, the distance of PA to PCL center is 29 mm [27]. It is therefore highly recommended to perform arthroscopy involving the posterior recessus with the knee in 90° of flexion.

**Fig. 3:**
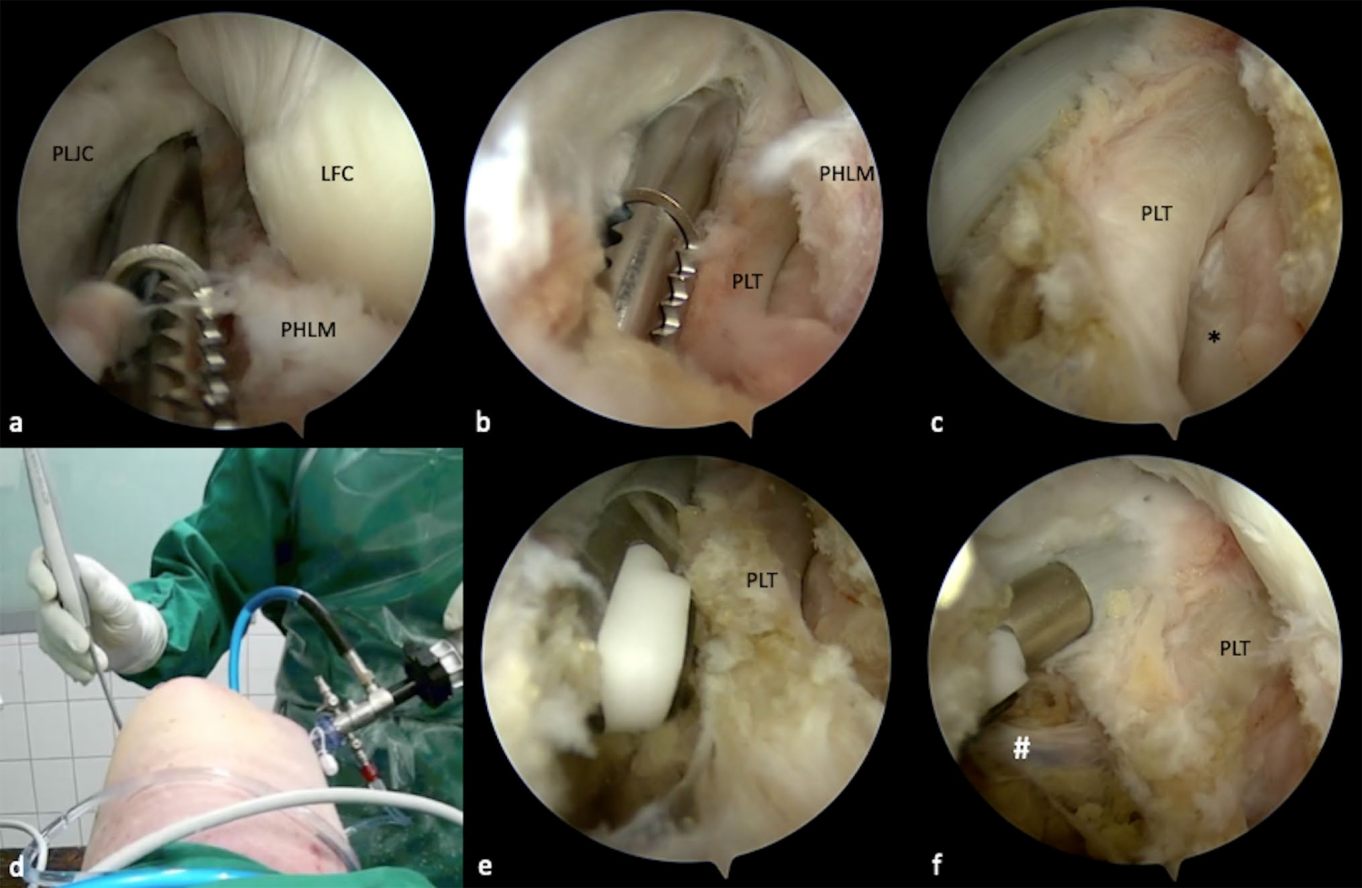
Preparation of the popliteus muscle–tendon unit. With a shaver, the popliteomeniscal fibers are carefully resected, in order to expose the popliteus tendon (PLT) while preserving the lateral meniscus (**a**). The PLT is located closely underneath the fibers, inside the popliteal sulcus (black asterisk) (**b**, **c**). In case of a popliteus bypass, this view is essential for tibial tunnel placement. Further preparation is performed closely to the popliteus muscle, to avoid injury of neurovascular structures. Thus, a radiofrequency electrode (CoolCut®, Arthrex, Naples, FL, USA) can be utilized, to prevent excessive bleeding (**d**, **e**). The lateral inferior genicular artery (LIGA) (white hash) is a frequent cause of bleeding (**f**). It is located at the dorsolateral side of the popliteus tendon, behind the soleus muscle and should be spared or coagulated to prevent bleeding. *PLJC* posterolateral joint capsule, *LFC* lateral femoral condyle, *PHLM* posterior horn of the lateral meniscus, *PLT* popliteus tendon
  The fibular head (FH) can typically be palpated distally and laterally to the PLT [15]. After intraarticular palpation, the posterolateral joint capsule directly dorsal to the PLT is carefully removed with a shaver, while the posterior edge of the FH should not be exceeded (Fig. 4a). Repetitive external palpation can facilitate the process of locating the FH. The FH presents one of the key landmarks for arthroscopic PLC reconstruction and should be thoroughly exposed (Fig. 4b).

- Step 4: Posterolateral portal and the popliteal muscle–tendon unit
  Under direct visualization of the posterolateral joint capsule through the PM portal, a needle is inserted in the safe triangle between the fibular FCL insertion, the lateral femoral epicondyle and the anterior boarder of the biceps femoris tendon. With the needle, the accessibility of all structures is validated to avoid anterior misplacement. Then, the PL portal is created with a blunt stab incision, located strictly anterior of the palpable biceps femoris tendon to avoid injury of the peroneal nerve. Furthermore, needle placement proximal/dorsal to the lateral femoral condyle and dorsal to the popliteus tendon in 90° knee flexion prevents injury of the FCL. With a shaver or a radiofrequency (RF) electrode, the popliteomeniscal fibers at the hiatus popliteus can be carefully resected for further exposition the popliteus tendon (PLT) and the popliteal muscle tendinous junction from a dorsal view (Fig. 3a, b). Subsequently, retraction of the PLT exposes its tibial sulcus. Anterior of the popliteal muscle–tendinous junction, the tibial drill tunnel exit of an arthroscopic popliteus bypass is located (Fig. 3c) [13]. Further preparation should be oriented dorsal of the popliteus muscle (Fig. 3d, e). The lateral inferior genicular artery (LIGA) is located dorsally of the popliteus tendon and laterally of the soleus muscle, with the potential of iatrogenic injury. Cauterization with an RF electrode prevents severe bleeding and an arthroscopic “red out”, without subsequent complications [28] (Fig. 3f).

**Fig. 4:**
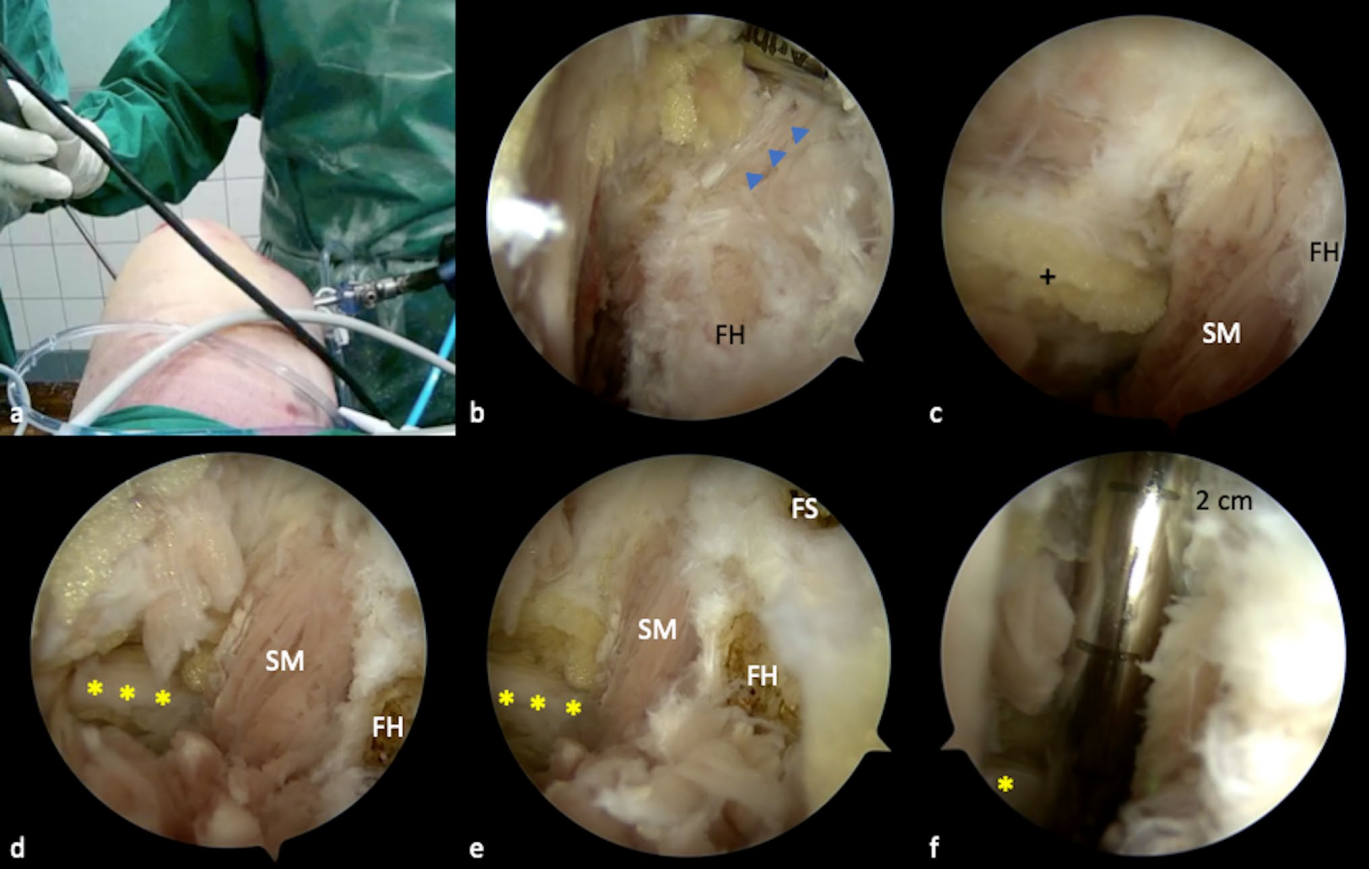
Exposure of the fibula head and the peroneal nerve. For preparation of the fibula head, a less traumatic shaver blade is advantageous (Torpedo®, Arthrex, Naples, FL, USA) (**a**). The fibula head can be palpated through the skin, to generate observable intraarticular movement. After careful preparation, the popliteofibular ligament (blue triangles) appears as a broad and fan-like shiny structure, which originates distally and dorsally of the styloid process and inserts at the musculotendinous junction of the PLT (**b**). Fat tissue (black cross), which is located dorsally of the soleus muscle indicates the course of the peroneal nerve (**c**). After careful resection, the nerve (yellow asterisks) can be visualized (**d**). In relation to the fibula head, the nerve is located lateral and distal to the hypothetical direction of a fibular drill channel for PLC reconstruction (**e**). From the tip of the styloid process to the peroneal nerve, a distance of 2.5 cm is measured (**f**). *FH* fibula head, *SM* soleus muscle, *SM* soleus muscle, *FS* fibular styloid
  The popliteofibular ligament (PFL) can be visualized after careful removal of surrounding soft tissue from the PLT (Fig. 5a). It originates at the tip of the dorsomedial fibular styloid and inserts at the popliteal muscle tendinous junction (Fig. 6) [29]. Arthroscopically, it can be identified as a short, fan-like and reflective ligamentous structure (Fig. 4b).
- Step 5: The peroneal nerve
  **Fig. 5:**
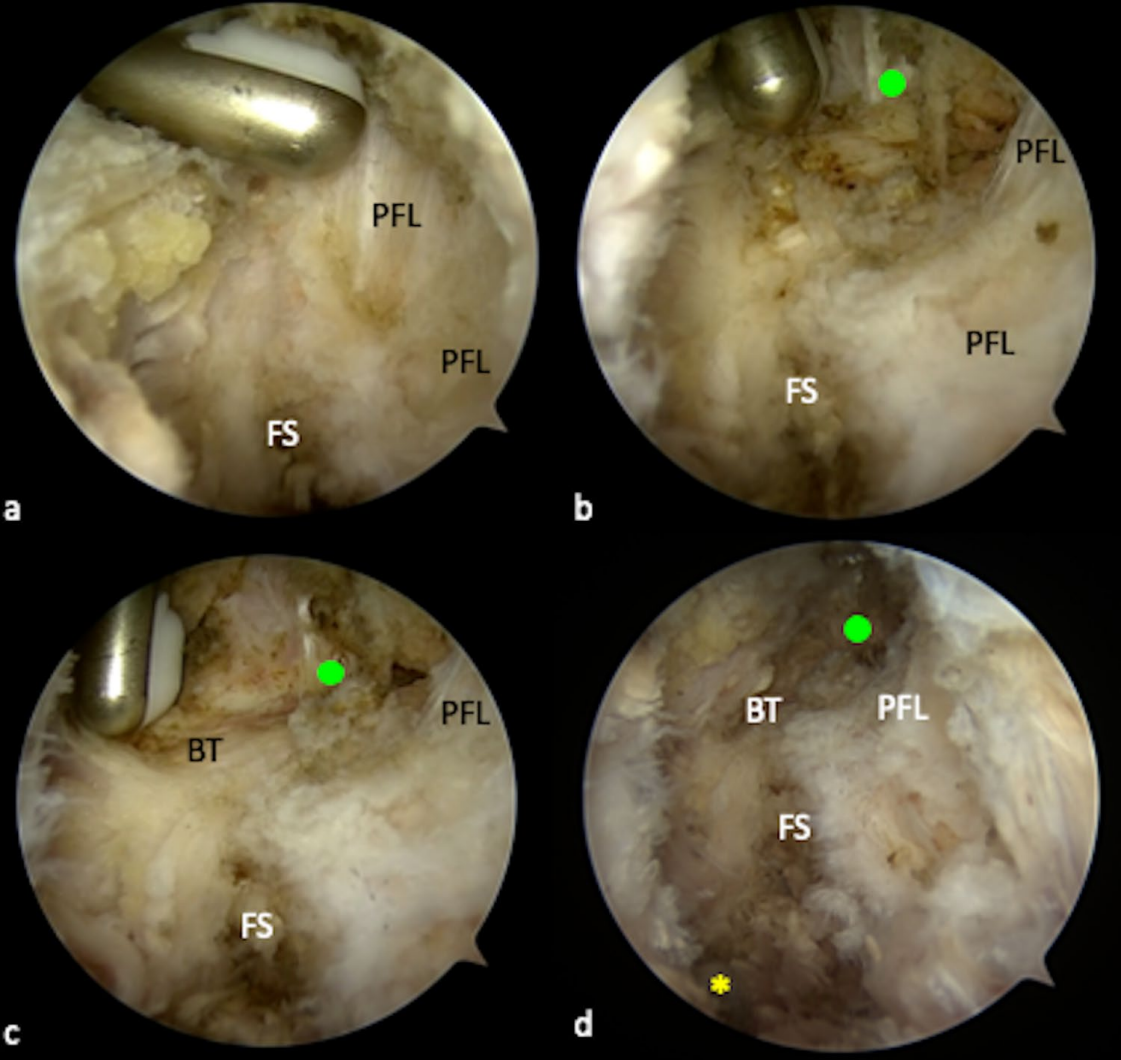
Preparation of the lateral collateral ligament (FCL). The fibular FCL attachment is partially covered by the posterior parts of the popliteofibular ligament (PFL) (**a**). Therefore, the most dorsal fibers need to be removed, in order to gain access to the FCL (**b**). The FCL appears as a shiny vertical structure, at the lateral side of the fibula head (green dot). Inferior and posterior to the FCL, the horizontal course of the biceps femoris tendon (BT) is visible (**c**). Although neither the FCL, nor the peroneal nerve (yellow asterisk) should be arthroscopically exposed in patients, the anatomic locations of both structures in relation to the fibula head are important to know when performing arthroscopic PLC reconstruction (**d**). *FS* fibular styloid, *PFL* popliteofibular ligament, *BT* biceps femoris tendon

**Fig. 6:**
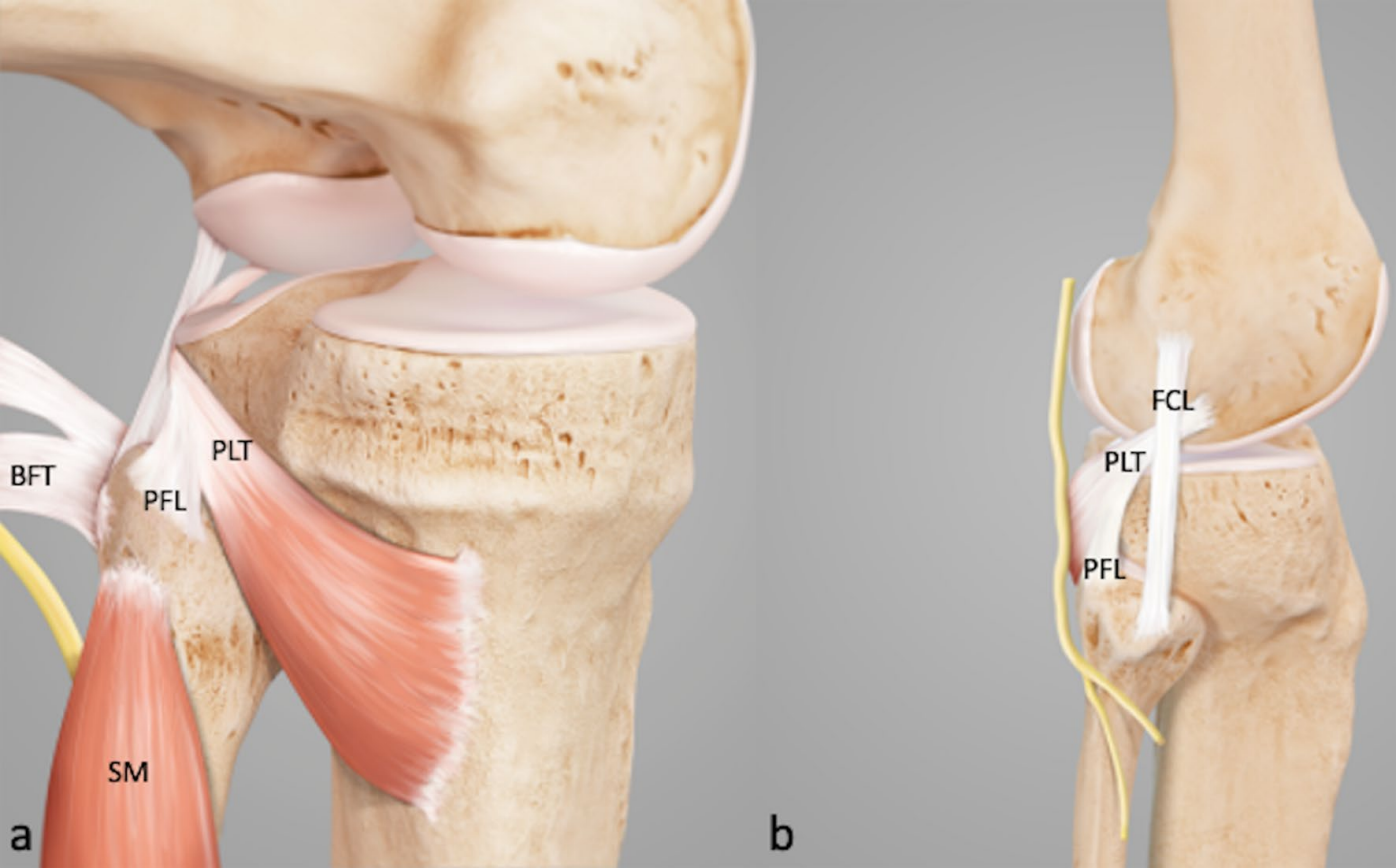
Annotated illustration of the key structures in the PLC (**a**). Location of the peroneal nerve in the PLC (**b**). *PLT* popliteus tendon, *PFL* popliteofibular ligament, *BFT* biceps femoris tendon, *SM* soleus muscle, *FCL* fibular collateral ligament
  Arthroscopic neurolysis puts the peroneal nerve at great risk of injury. Although neurolysis is mandatory when performing open-surgical PLC reconstruction, it is not recommended for arthroscopic techniques [19]. Nevertheless, the precise knowledge of anatomical relations is essential, particularly when performing arthroscopic PLC reconstruction techniques (Fig. 6b). Because the peroneal nerve normally remains unseen, the anatomic landmarks should be visualized properly and special attention must be given to the angulation and height of the fibular drill channel. This way, the risk of injuring the peroneal nerve can be reduced [15, 19]. In the course of this study, the peroneal nerve is exposed only for demonstration purposes. The posterolateral joint capsule along the biceps femoris tendon can be carefully resected at about 2–3 cm distal to the fibular styloid, dorsal of the fibula and lateral of the soleus muscle. Fatty tissue (perineural fat) at the dorsolateral side of the soleus muscle, below its most proximal fibular insertion, indicates the location of the peroneal nerve (Fig. 4c). After resection of the capsular fibers and perineural fat tissue, the peroneal nerve can be visualized approximately 2–3 cm distal to the fibular styloid (Fig. 4d–f).
- Step 6: Preparation of the fibular collateral ligament (FCL)
  The femoral attachment and the distal portion of the FCL can also be arthroscopically exposed. For the femoral footprint an additional lateral parapatellar portal is necessary. At the lateral femoral epicondyle a layer of soft tissue is gently resected, as described before, to expose the femoral FCL and PLT attachments [15]. The fibular attachment is located outside the joint capsule, on the lateral side of the fibula head and distal and anterior to the styloid process and PFL attachment [29]. It is partially covered by the tendon of the biceps femoris muscle (BT) and by the most dorsal fibers of the popliteofibular ligament (PFL) [30] (Fig. 5a). To expose the FCL, the most dorsal parts or the PFL and the lateral joint capsule must be removed (Fig. 5b). With the arthroscope still placed in the PM portal, it can be helpful to follow the course of the PLT, which crosses under the FCL (Fig. 6). The FCL appears as a shiny cord-like ligament structure at the lateral side of the fibular head (Fig. 5c, d).

## Discussion

Arthroscopic treatment of posterolateral instabilities has shown an increasing popularity in the recent literature [13–18]. However, although the recent studies have described convincing biomechanical and clinical results, open-surgical procedures are still the preferred treatment of most surgeons for posterolateral instabilities [14, 18] (Table 1). The underlying concerns mainly arise from an uncertainty regarding anatomic relations of the PLC, during arthroscopic visualization. Recently, Chahla et al. have pointed out several limitations of arthroscopic interventions near or at the PLC. Among others, a limited exposure of anatomic landmarks with subsequent non-anatomic tunnel (mis-) placement and an increased risk of neurovascular injuries are proposed disadvantages [19], which is not reflected by our experience and our anatomic and clinical studies [2, 11, 13, 15, 28] (Table 2).

**Table 1:** Pearls and pitfalls of posterolateral preparation

	Pearls	Perils and Pitfalls
Posteromedial (PM) portal	Needle probing under transcondylar visualization allows targeted preparation of transseptal approach	Anterior misplacement can lead to restriction of instruments during posterolateral preparation (high risk to occur)
	Inserting a cannula into the portal can facilitate portal preservation and navigation of instruments during preparation	
Posterolateral (PL) portal	Needle insertion and stab incision should be performed after establishment of a transseptal approach with subsequent direct visualization in order to prevent portal misplacement	Misplacement of PL portal results in improper preparation and exposure of relevant structures Placement too far anteriorly can cause injury to the FCL (high risk to occur)
	Needle probing can ensure proper accessibility of all posterolateral structures	
Resection of popliteomeniscal fibers	A less traumatic shaver can be used to open the popliteomeniscal fibers. Further preparation can be performed with a RF electrode, in hook-in technique	Rash resection or improper electrocautery can easily lead to injury of the PLT or the PFL (intermediate risk to occur)
Transseptal approach	High transcondylar approach between ACL and PCL allows safe preparation of dorsal septum	Low position of transseptal portal due to low transcondylar view during septum preparation
	Indirect transcondylar visualization of dorsal septum from the lateral side allows precise preparation of transseptal portal However, extensive scarring in revision surgery may require visualization from posteromedial	Damage to PCL fibers or popliteus muscle fibers caused by low septum preparation during preparation under direct visualization from posteromedial (high risk to occur)
	High or low placement of transseptal portal can facilitate visualization of targeted structures above or below popliteomeniscal fibers	Resection of the posterior joint capsule during septum preparation can cause vascular injury (intermediate risk to occur)
	Ventral orientation of the shaver blade and staying close to the bone prevents dorsal misguidance of preparation	
	Storing the knee in 90° of flexion leads to a more dorsal placement of the popliteal artery to prevent injury	
Fibular head preparation	External palpation of fibular head allows its precise location but might be limited	Preparation posterior to fibular head may increase the risk of injury to the inferior lateral genicular artery or the peroneal nerve (high risk to occur)
	Keeping the knee bent in 90° of flexion during preparation and staying proximal reduces the risk of peroneal nerve injury

**Table 2:** Clinical outcomes and complications of posterolateral arthroscopy and implementation of a transseptal approach

Author and year	Study design	Number of cases	Follow-up	Indication	Outcome	Complications
Frosch et al. 2016	Retrospective case series	*n* = 12	1 year	Anatomic popliteus bypass + PCL reconstruction	Lysholm Score: 88.6 ± 8.7 Posterior drawer test (SSD): 13.3 ± 1.9 mm (preoperative) 2.9 ± 2.2 mm (postoperative)	None
Song et al. 2015	Case report	*n* = 1	2 years	Fibula-based PFL reconstruction	Posterior drawer test (SSD): 11.8 mm (preoperative) 3.8 mm (postoperative) External rotation in dial test (SSD): 16° (preoperative) 4° (postoperative)	None
Chen et al. 2018	Retrospective cohort study	*n* = 76	3 weeks	Removal of loose bodies	Lysholm Score: 74.8 ± 4.2 (preoperative) 92.8 ± 3.7 (postoperative Tegner Score: 2.9 ± 0.87 (preoperative) 5 ± 0.96 (postoperative) IKDC Score: 56.4 ± 3.9 (preoperative) 75.4 ± 4.7 (postoperative)	Not reported
Ohishi et al. 2015	Retrospective cohort study	*n* = 161	Not reported	Synovectomy *n* = 57 Meniscal resection *n* = 20 Thermal shrinkage *n* = 1 Resection of PCL ganglion *n* = 1 Meniscal cyst decompression *n* = 1 Popliteal cyst decompression *n* = 53 Repair of PCL avulsion fracture *n* = 5 Free body resection *n* = 5 Repair of posterior horn of the medial meniscus *n* = 5 PCL reconstruction *n* = 2 Probing only *n* = 11	Not reported	*n* = 4 (2.5%) Superficial infection of PM portal (*n* = 2) Subcutaneous hematoma (*n* = 1) Deep infection of PL portal (*n* = 1)
Kyung et al. 2012	Case report	*n* = 2	16 months, 24 months	Resection of synovial chondromatosis	Case 1: pain free, full range of motion, light sport activity Case 2: pain free, full range of motion	None
Herode et al. 2016	Case report	*n* = 1	10 months	Resection of medial meniscal cyst	Pain free, no recurrence	None

In open-surgical anatomic PLC reconstruction techniques, fibular tunnel placement is based on manual palpation, as the dorsomedially located PFL attachment cannot be visualized. Furthermore, its anatomic footprint does not comply with the location of the drill channel [29, 31]. In contrast, arthroscopic approaches might allow an exact tunnel placement, under direct visualization of fibular, femoral and tibial attachment sites [15, 17]. Accordingly, Frosch et al. have observed a high accuracy and reproducibility of tunnel placement for arthroscopic popliteus-bypass reconstruction [2]. In the presented article, all relevant landmarks necessary for complex posterolateral reconstruction could be prepared and exposed arthroscopically, respectively. Thereby, the peroneal nerve was shown to be located at about 2–3 cm distally to the fibular styloid. Although arthroscopic exposure of the peroneal nerve is not recommended in actual patients, the necessary extent of preparation and its observed distance from the fibular insertion sites show that direct visualization of the nerve during fibular drill channel placement is not necessary for arthroscopic posterolateral corner reconstruction. This is particularly true, if the intended drilling direction from anterolateral-distal to posteromedial-proximal is obeyed.

Arthroscopic visualization of the fibular FCL attachment was performed for educational purposes and should also not be performed in PLC reconstruction. Accordingly, the mandatory stab incision over the fibular head for LCL tunnel placement makes arthroscopic visualization abdicable.

An important aspect of this article is the use of a transseptal posteromedial approach, which is crucial in order to fully visualize the posterolateral corner. With the use of the transseptal approach, the entire PLC can be visualized and accordingly addressed. A direct lateral view on the dorsal septum during preparation of the approach is helpful to ensure proper placement and prevent accidental injury of PCL fibers or misguidance.

Generally, the arthroscopic procedures are known to bear a considerable learning curve. Although this has been concisely demonstrated for standard arthroscopic approaches, the presented technique for posterolateral arthroscopy certainly requires a fair amount of arthroscopic experience, in order to allow anatomic orientation and to reduce the complication rate [32, 33]. Therefore, posterolateral arthroscopy represents a suitable option for the experienced arthroscopic surgeon [15–17].

In conclusion, this article provides an arthroscopic overview of the PLC, with regard to important anatomic structures and drill channel placement. With the knowledge of their location and under continuous visualization, arthroscopic complex PLC reconstruction becomes a precise and safe procedure.

## Supplementary Information

Below is the link to the electronic supplementary material.

Supplementary file 1 (MP4 446580 KB)
